# *Onosma anatolica*, a new species of Boraginaceae from Turkey

**DOI:** 10.3897/phytokeys.69.8360

**Published:** 2016-08-25

**Authors:** Rıza Binzet

**Affiliations:** 1 Mersin Üniversity, Faculty of Arts and Sciences, Department of Biology, 33343, Mersin, Turkey

**Keywords:** Boraginaceae, Onosma, Endemic, Niğde, Turkey

## Abstract

*Onosma anatolica* Binzet, is described and illustrated as a new species from Niğde province in southern Anatolia, Turkey. It belongs to sect. Onosma L. subsect. Asterotricha (Boiss.) Gürke. The new species is closely related to *Onosma subulifolia* Riedl from which it is distinguished. *Onosma anatolica* is readily distinguished from *Onosma subulifolia* by its sterile shoots, the green-grey stem indumentum, longer bracts, yellow and puberulous petals. The IUCN threat category of *Onosma anatolica* is determined as “CR (Critically Endangered)”. A distribution map and anidentification key for *Onosma anatolica* and *Onosma subulifolia* supplement the study.

(Critically Endangered)

## Introduction

The family Boraginaceae s.s. comprise some 1600 species (Chacon et al. 2016, Luebert et al. 2016 Mehrabian et al. 2012, Kolarčik 2010; Cecchi and Selvi 2009). Members of the family are widespread in western and central Asia and in the Mediterranean area (Jávorka 1906, Meusel et al. 1978). The family comprises about 44 genera and 375 species in Turkey (Güner 2012).

The genus *Onosma* L. (Boraginaceae-Lithospermeae) is one of the largest in the Boraginaceae and includes about 230 species, mainly distributed in the Mediterranean region, southwest Asia, and temperate Europe (Boissier 1879, Riedl 1967, Peruzzi and Passalacqua 2008, Binzet et al. 2010, Mehrabian et al. 2011, 2014, Ranjbar and Almasi 2014). In Turkey, the genus is represented by about 103 species as the rate of species endemism amount to 50% (Riedl 1978, Davis et al. 1988, Yıldırımlı 2000, Riedl et al. 2005, Binzet and Orcan 2007, Kandemir and Türkmen 2010, Aytaç and Türkmen 2011, Koyuncu et al. 2013, Guner 2012, Binzet 2016). This genus is perennial, usually suffrutescent or consists of biennial herbs (Stevanovic et al. 2003, Akçin and Binzet 2011). *Onosma* is systematically difficult, and most of the diagnostic characters are based mainly on the whole indumentums, leaf and flower morphology (Ball 1972, Riedl 1978, Peruzzi and Passalacqua 2008, Pavlova 2009). To identify the sectional categories of the *Onosma* members, the indumentum of the stem and the leaves are of significance (Özcan 2009). The genus has been divided into three groups, originally described as sections, but here recognized only as informal groups: *Asterotricha*, with basal leaves covered by stellate trichomes or asterosetae (i.e. along tubercled seta with several shorter rays at its base), *Haplotricha*, with basal leaves covered by simple setae, and *Heterotricha*, with both simple setae and asterosetae on the basal leaves (Boissier 1879, Peruzzi et al. 2004, Peruzzi and Passalacqua 2008, Koyuncu et al. 2013).

During a field trip in June 2011, some interesting *Onosma* specimens were collected from the Niğde province in Southern Anatolia. The few plants gathered in 2011 were complemented by collection conducted in June 2015 from the same site. Detailed morphological studies indicated that the population of *Onosma* from the Niğde province represent a hitherto unknown species related to *Onosma subulifolia*. This species is described here as new to science bringing the total number of the species known from Turkey to 104.

## Material and methods

Specimens of *Onosma anatolica* were collected by the author during three field excursions in Niğde province in June 2011 and June 2015. Totally 8 herbarium specimens were collected from one locality and deposited in ANK, GAZI and the Herbarium of Mersin University. I have compared the new *Onosma anatolica* specimens with *Onosma subulifolia* based on relevant taxonomic literature (Riedl 1978). The holotype photo of *Onosma subulifola* was taken from E. Preliminary conservation assessments were made using the IUCN (2012) guidelines. For the palynological definision in total 50 pollen grains and 20 mature nutlets were measured using a light microscope (LM) and stereo-binocular microscope. In addition, observations were made using a scanning electron microscope (SEM).

For pollen studies using LM, grains were taken from fresh and herbarium materials prepared according to the Wodehouse methods (Wodehouse 1935). The polar axis (P), equatorial axis (E), and other characteristics (plg, plt, clg, clt, exine, intine and t; see Table 1 for abbreviations) of the pollen grains were measured using an Olympus BX40 with a 100× objective until a Gaussian curve was acquired (Table 1). For SEM observations, pollen grains obtained from each specimen were transferred onto stubs and coated with platinum. The SEM micrographs were taken with a ZEISS supra 55. In this study, nomenclature for pollen morphology was used in accordance with Wodehouse (1935), Faegri and Iversen (1989) and Punt et al. (1994).

**Table 1.** Pollen morphological parameters of *Onosma anatolica* (W: Wodehouse Method).

Taxa	Pollen shape P/E	P [µm]	E [µm]	plg [µm]	plt [µm]	clg [µm]	clt [µm]	Ex [µm]	i [µm]	t [µm]
*Onosma anatolica*	prolate 1.35	17.31	12.76	2.90	3.02	12.76	2.95	0.70	0.50	5.93

P: length of the polar axis; E: width of the equatorial axis; plg: length of the pores (pori); plt: width of the pores (pori) clg: length of the colpus (colpi); clt: width of the colpus (colpi); Ex: exine thickness; i:intine thickness; t: length of the polar triangular edge.

Morphological characters of *Onosma subulifera* used in this paper for comparation were obtained from relevant taxonomic literature (Riedl 1978).

## Results

### Onosma anatolica (Sect. Onosma subsect. Asterotricha)

Taxon classificationPlantaeBoraginalesBoraginaceae

Binzet sp. nov.

urn:lsid:ipni.org:names:60472943-2

Figures 1 , 2

#### Type.

Turkey, C5 Niğde: Çamardı, 2 km South of Demirkazık village, subalpine community with dwarf shrub and thorn-cushion, 1760 m, limestone, 22 June 2015, 37°50'47"N, 35°05'32"E, *Binzet 201501* (holotype: ANK; isotype: GAZI).

#### Diagnosis.

*Onosma anatolica* is related to *Onosma subulifolia* especially by its habit, calyx and corolla length. However, the new species differs mainly in its sterile shoots; a green-grey stem with adpressed setose trichomes, the setae arising from short stellate hairy and shortly hairy (not bluish-black, otherwise glabrous as in *Onosma subulifolia*); sterile rosette leaves and basal leaves -50 × 1–1,5(-1.8) mm, revolute-subulate; cauline leaves -20 × 1–1.5(-1.8) mm, (as opposed to 15–32 × 1.5(-1.8) mm in *Onosma subulifolia*); an inflorescence of 1–2 cymes, sometimes elongated after flowering (not short subcapitate); flower bracts 8–15 × 1–2 mm, the apex acute (as opposed to 7–8 mm, apex lingulate in *Onosma subulifolia*); pedicel 1–2 mm (not 1–1.5 mm); calyx 6–8 mm in flower, 8–12 mm in fruit, suboblate in shape, patent strigose on ±tubercles and shortly hairy on the outside and rarely hairy on the inside (as opposed to 10–11 mm, subobtuse, and hispid in *Onosma subulifolia*); corolla yellow, clavate, puberulous, lobes 1 mm long, 1.7 mm wide at base, widely triangular (as opposed to white, clavate-campanulate, glabrous, lobes short, acute in *Onosma subulifolia*); and nutlets 3–3.5 × 2–2.3 mm, short beaked, grey.

**Figure 1.**
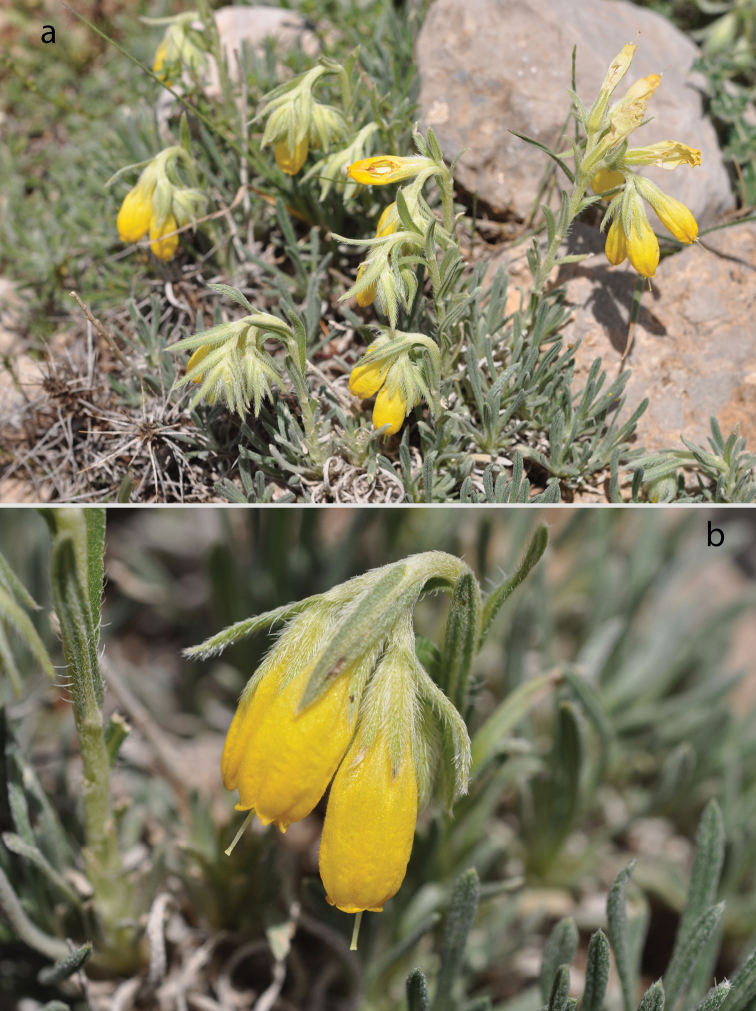
In situ photographs of *Onosma anatolica* (**a** habit **b** flowers).

**Figure 2.**
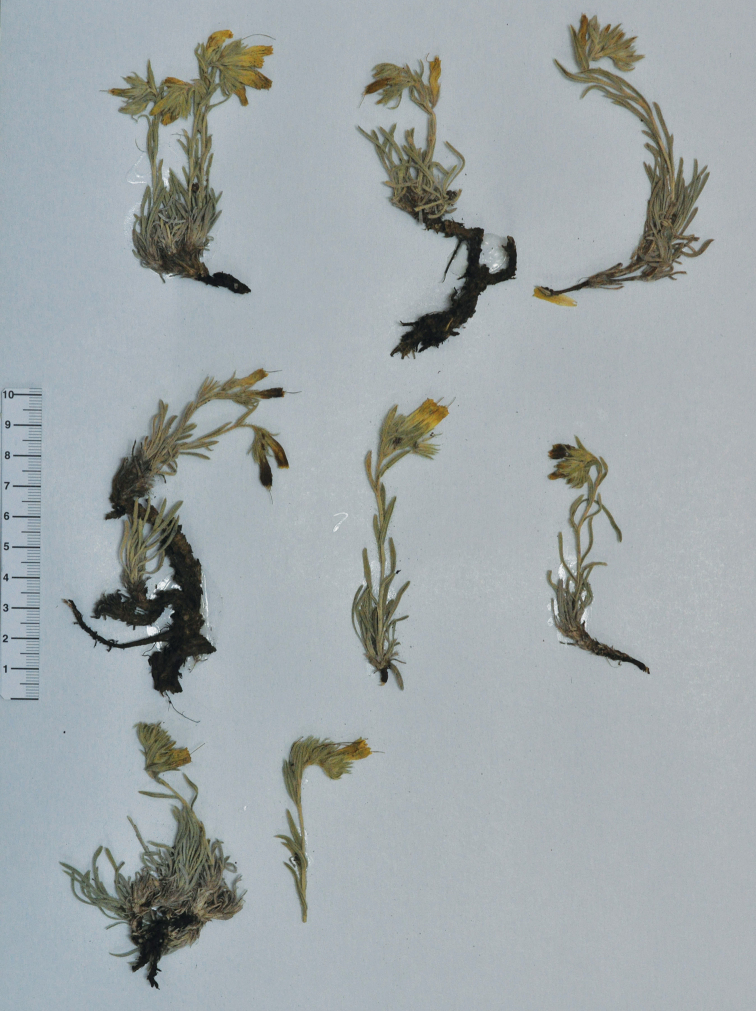
The holotype of *Onosma anatolica* from the herbarium ANK: *Binzet 201501*, 22 June 2015. (Photo Rıza Binzet).

#### Description.

Perennial rhizomatous herb, rhizome divided into several, ca. 8 mm diameter, subterranean, creeping branches. Aerial stems up to 12 cm tall(including inflorescence), ca. 1(-1.5) mm in diameter, green to grey, covered by adpressed setose, setae arising from short stellate trichomes, shortly hairy, sterile rosette to 5 cm. Leaves crowded at base, leaves of the sterile rosette and basal leaves -50×1–1.5 (-1.8) mm, acute, revolute-subulate, adpressed setose, setae arising from stellate trichomes (asterotrichous state). Cauline leaves -20×1–1.5(-1.8) mm, acute, indistinct revolute-subulate, upper surface covered by densely adpressed setae arising from stellate hairy and sparsely adpressed setose, setae arising from stellate hairy on lower surfaces. Upper cauline leaves resembling lower ones, but smaller in size. Inflorescence of 1–2 cymes, forming a short subcapitate cluster, sometimes elongating after flowering. Flower bracts 8–15 × 1–2 mm, linear-subulate, abruptly narrowed and tapering towards an acute apex, ± patent setose with stellate trichomes and tubercles. Pedicels 1–2 mm. Calyx 6–8 mm in flower, 8–12 mm in fruit, free to base, lobes narrowly linear, suboblate densely covered with patent strigose on ± tubercles and shortly hairy on the outside and rarely hairy on the inside. Corolla yellow, 15–18 mm, clavate, puberulous, reflexed lobes 5, 1 mm long, 1.7 mm wide at base, widely triangular, acute, annulus glabrous. Anthers included, linear, ca. 6 mm, sagittate, connate at base, included or sterile tips exerted. Filaments ca. 5 mm. Style 3–5 mm protruding outside the corolla limb, stigma small, distinctly bilobed. Nutlets 3–3.5×2–2.3 mm, shortly beaked, grey. Pollen grains heteropolar, shape prolate P/E(Polar axis/Equatorial axis) ratio 1.35.

#### Etymology.

The species epithet *anatolica* refers to Anatolia, the Asian part of Turkey.

#### Distribution and ecology.

*Onosma anatolica* is distributed in southern Anatolia (Niğde) and grows in subalpine dwarf shrub and thorn-cushion communities. The geological substrat is limestone and the new species occurs only between 1700 and 1800 m. The species belongs to the Irano-Turanian floristic region. Species growing in close proximity to the vew species are: Astragalus angustifolius Lam. subsp. angustifolius, *Euphorbia kotcchyana* Fenzl. *Bromus cappadocicus* Boiss. et Bal. *Marrubium heterodon* (Benth.) Boiss. & Balansa, *Poa bulbosa* L., *Bromus tectorum* L., *Centaurea pseudoreflexa* Hayek, *Centaurea chrysantha* Wagenitz, *Astragalus* sp. *Sideritis libanotica* Labill. subsp. *Linearis* (Bentham). Bornm., *Anthemis* sp., *Galium incanum* SM., *Convolvulus compactus* Boiss., and *Alyssum aureum* (Fenzl.) Boiss. Phytosociologically the community where the new species occurs can be grouped into Astragalo-Brometea Quézel 1973 class and Astragalo-Brometalia Quézel 1973 order (Quézel 1973, Eren et al. 2004, Parolly 2004).

#### Conservation status.

*Onosma anatolica* is hitherto known only from the type locality, and its estimated area of occupancy is less than 50 km^2^. Moreover, the area is subjected to heavy grazing pressure. Because of its localized distribution, small population size and grazing pressure, it should be considered as ‘Critically Endangered (CR)” according to the IUCN criteria A3 and B2 (IUCN 2012).

#### Additional specimens (paratype).

Type locality, 10 June 2011, Binzet 201122 (Mersin University).

#### Additional specimens examined.

***Onosma subulifolia***: Turkey A5 Sinop: after Kargı, 250 m, *Tobey 2625*, 11 May 1969 (holotype, E 00022534, photo).

#### Phenology.

Flowering from May to June, setting fruit until late July.

#### Palynology.

Pollen grains are heteropolar, trisyncolporate and subprolate. Exine surface of the grains is insular. The insulae have free scabrae and the scabrae are widely spaced. The average means of the number of scabrae in each insulae range from 6 to 15. The main palynological characters and SEM micrographs of *Onosma anatolica* are presented in Table 1 and Figure 5.

#### Nutlet morphology.

Nutlet size shows some variations, Nutlets 3–3.5×2–2.3 mm, shortly beaked, and grey. Nutlet surface ornamentation is rugose and characterized by the epidermal cells of the nutlet surface having small or fine wrinkles (Fig. 6).

**Figure 3.**
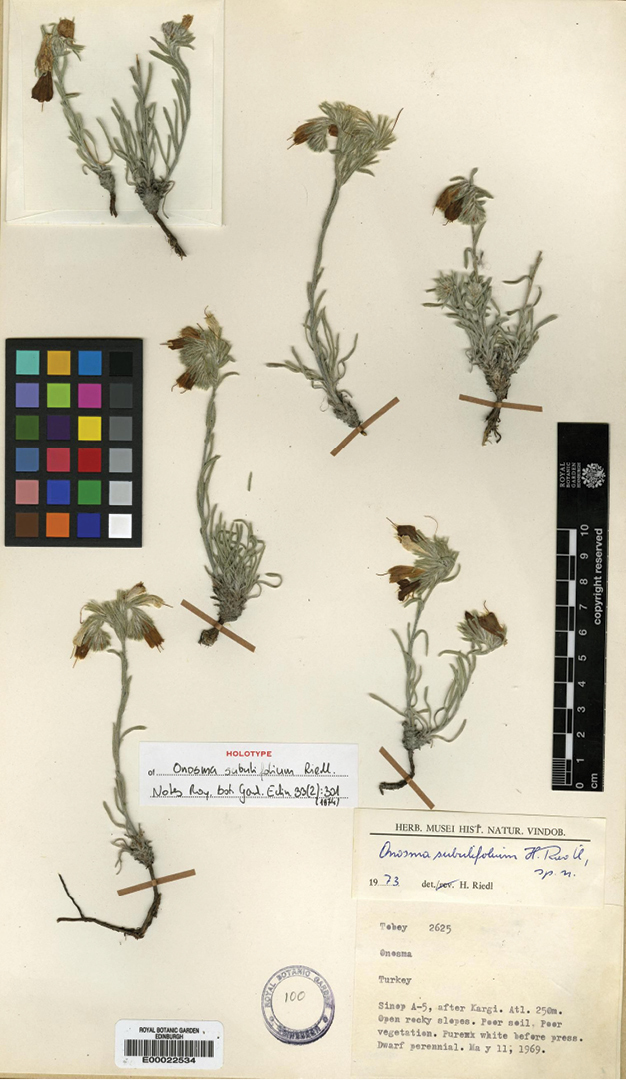
The holotype of *Onosma subulifolia*: Tobey 2615, 11 May 1969 (E 00022534); image available at website: Source: http://elmer.rbge.org.uk/bgbase/vherb/bgbasevherb.php

**Figure 4.**
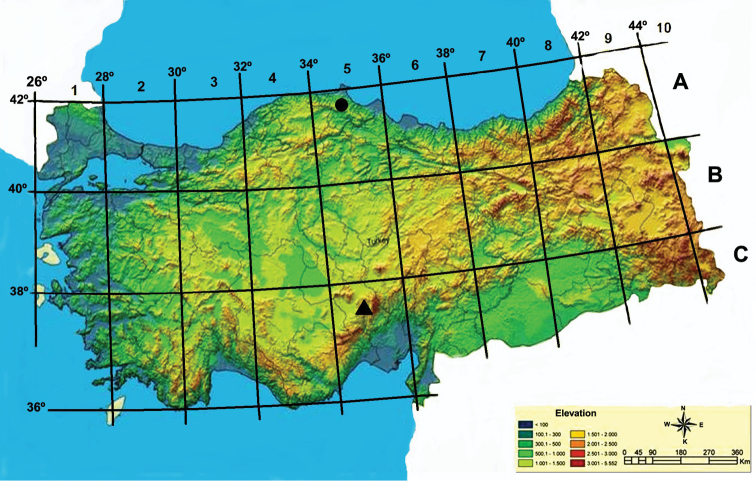
Distribution map of *Onosma anatolica* Binzet (filled triangle) and *Onosma subulifolia* (filled circle).

**Figure 5.**
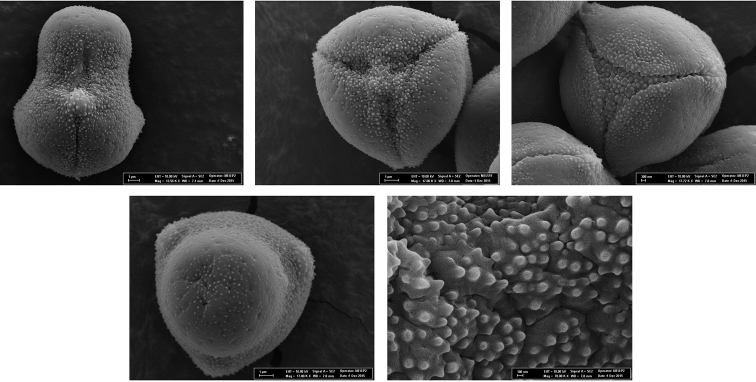
SEM microphotograph of pollen of *Onosma anatolica*.

**Figure 6.**
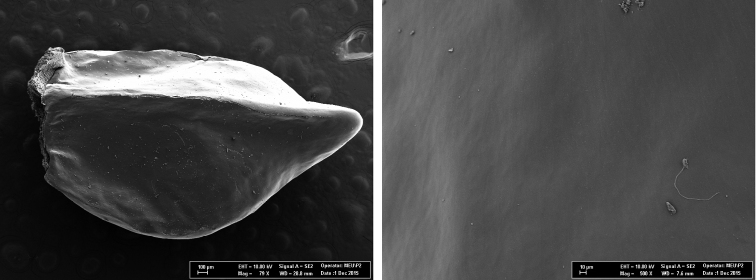
SEM microphotograph of nutlet of *Onosma anatolica*.

### An updated key to the several flowering stems and sterile rosettes, leaves 1–1.5(-1.8) mm broad species of *Onosma* (*Asterotrichous* group) in Turkey is proposed below.

**Table:** 

1	Bracts 7–8 mm, calyx 10–11 mm, corolla white, clavate-campanulate, glabrous.	***Onosma subulifolia***
–	Bracts 8–15 mm, calyx 6–12 mm, corolla yellow, clavate, puberulous	***Onosma anatolica***

## Discussion

*Onosma anatolica* belongs to Onosma sect. Asterotricha subsect. and it is distributed in Southern Anatolia (Niğde) and grows on the steppe and rocky pastures. It is an element belong to the Irano-Turanian floristic region. It shows some affinity to *Onosma subulifolia* which is placed in the same subsection and can be easily distinguished from that species by its sterile shoots; green-grey stem with adpressed setose trichomes, the setae arising from stellate hairy and shortly hairy, sterile rosette leaves and basal leaves -50 × 1–1.5(-1.8) mm; revolute-subulate, cauline leaves -20 × 1–1.5(-1.8) mm; bracts 8–15 × 1–2 mm; calyx 6–8 mm in flower, 8–12 mm in fruit, suboblate, patent strigose on ±tubercles and shortly hairy on the outside, rarely hairy on the inside; yellow corolla, clavate, puberulous, nutlets 3–3.5 × 2–2.3 mm in size, short beaked, grey. Details of the differences between *Onosma anatolica* and *Onosma subulifolia* are presented in Table 2.

**Table 2.** The morphological differences between *Onosma anatolica* and *Onosma subulifolia*.

	*Onosma subulifolia*	*Onosma anatolica*
Stem	Bluish-black, adpressed setose, otherwise glabrous.	Green-grey, woody at base, adpressed setose with stellate hairy and shortly hairy.
Leaves	15–32 × 1–1.5(-1.8) mm, involute-subulate, densely adpressed setose.	Sterile rosette and basal leaves -50 × 1–1.5(-1.8) mm, revulate-subulate, adpressed setose with stellate hairy. Cauline leaves -20 × 1–1.5(-1.8) mm, indistinct revulate-subulate, densely covered, adpressed setose with stellate hairs on upper surfaces and sparsely adpressed setose with stellate hairs on lower surfaces.
Inflorescence	Short subcapitate	Cymes 1–2, short subcapitate, sometimes elongated after flowering.
Bracts	7–8 mm, flat, apex lingulate.	8–15 × 1–2 mm, linear-subulate, abruptly narrowed and tapering towards an acute apex, ±patent setose, stellate and tuberculate.
Pedicel	-1–1.5 mm	1–2 mm
Calyx	10–11 mm, lobes narrowly linear, subobtuse, hispid.	6–8 mm in flower, 8–12 mm in fruit, lobes narrowly linear suboblate, patent strigose on ± tubercles and shortly hairy on outside and rarely hairy on inside.
Corolla	White, clavate-campanulate, glabrous, lobes short, acute.	Yellow, clavate, puberolous, lobes 1 mm long, 1.7 mm wide at base, widely triangular.
Nutlets	Unknown	3–3.5 × 2–2.3 mm, shortly beaked, grey.

## Supplementary Material

XML Treatment for Onosma anatolica (Sect. Onosma subsect. Asterotricha)
